# Machine learning-based model for predicting contralateral central lymph node metastasis in papillary thyroid carcinoma with isthmus proximity

**DOI:** 10.3389/fendo.2025.1728945

**Published:** 2026-01-09

**Authors:** Lin Wang, Yue Han, Chaohui Wang, Zhenhua Sun, Haitao Zhang

**Affiliations:** 1 Department of Thyroid and Breast Surgery, Affiliated Hospital of Jiangsu University, Zhenjiang, China; 2 Department of General Surgery, The First Affiliated Hospital of Soochow University, Suzhou, China

**Keywords:** contralateral central lymph node metastasis, isthmus proximity, machine learning, papillary thyroid carcinoma, predictive model

## Abstract

**Background:**

Papillary thyroid carcinoma (PTC) originating from the isthmus exhibits a marked tendency for contralateral central lymph nodes (Cont-CLNs) metastasis. To accurately assess this risk, this study aims to establish and validate an individualized predictive model for contralateral central zone lymph node metastasis in PTC with isthmus proximity using machine learning algorithms.

**Methods:**

This retrospective study analyzed 1,672 patients with PTC. Based on tumor location, patients were categorized into a group with PTC with isthmus proximity and a non-isthmic group to compare the incidence of Cont-CLNs metastasis. Subsequently, we focused on 397 patients with PTC with isthmus proximity, who were randomly allocated in a 7:3 ratio to a training set (n=279) and a validation set (n=118). Feature selection was performed using the Boruta algorithm and LASSO regression. Seven machine learning algorithms were then employed to construct prediction models. Model performance was evaluated using metrics including the AUC, sensitivity, and specificity. The optimal model was interpreted using the shapley additive explanations (SHAP) method.

**Results:**

This study included 1,672 patients with PTC. The rate of Cont-CLNs metastasis was significantly higher in patients with unilateral PTC with isthmus proximity (n=397) than in those with non-isthmic PTC (33% vs. 12%, *P* < 0.05). Feature selection using LASSO regression and the Boruta algorithm identified five key predictors: preoperative CT assessment, extrathyroidal extension, ipsilateral central lymph node (Ipsi-CLNs) metastasis, preoperative ultrasound assessment, and tumor size. Among the seven machine learning algorithms evaluated, the random forest model demonstrated the best overall performance, achieving the highest F1 score and AUC values of 0.942 in the training set and 0.861 in the validation set. SHAP interpretability analysis confirmed that preoperative CT assessment was the most influential predictor, and its impact pattern was highly consistent with established clinical knowledge.

**Conclusion:**

The machine learning model developed in this study effectively predicts the risk of Cont-CLNs metastasis in patients with unilateral PTC with isthmus proximity, providing a valuable tool to support personalized surgical decision-making regarding the extent of lymph node dissection.

## 1 Introduction

Thyroid cancer represents the most prevalent endocrine malignancy, with its incidence continuing to rise globally (1). Among its histologic subtypes, PTC is predominant, comprising 80–90% of all cases (2). Lymphatic spread is the principal metastatic route for PTC, and cervical lymph node involvement is frequently observed at initial diagnosis. The central compartment constitutes the primary nodal basin for metastatic dissemination (3). Current guidelines recommend prophylactic Ipsi-CLNs for high-risk unilateral PTC (4); however, Cont-CLNs metastasis may also occur (5). The necessity of routine Cont-CLNs dissection remains clinically controversial (6).

Clinical observation suggests that patients whose dominant tumor crosses the isthmus while remaining largely confined to one lobe may exhibit a higher incidence of Cont-CLNs metastasis compared to those with tumors in other locations—a finding supported by prior reports (7) and further validated in this study. Although bilateral central compartment dissection may improve locoregional control, it concurrently elevates the risk of permanent hypoparathyroidism and recurrent laryngeal nerve injury (8).

Consequently, determining the optimal extent of lymph node dissection for PTC with isthmic involvement poses a significant surgical dilemma. Given the paucity of systematic evidence on Cont-CLNs metastasis in this anatomic subtype, a reliable predictive tool is urgently needed. Machine learning methods, which excel in modeling complex clinical relationships, offer a promising approach. Therefore, this study aimed to develop and validate a machine learning−based model to predict Cont-CLNs metastasis in patients with unilateral PTC exhibiting isthmus proximity, thereby informing individualized surgical planning.

## 2 Materials and methods

### 2.1 Anatomical and radiographic definitions

**Figure 1:**
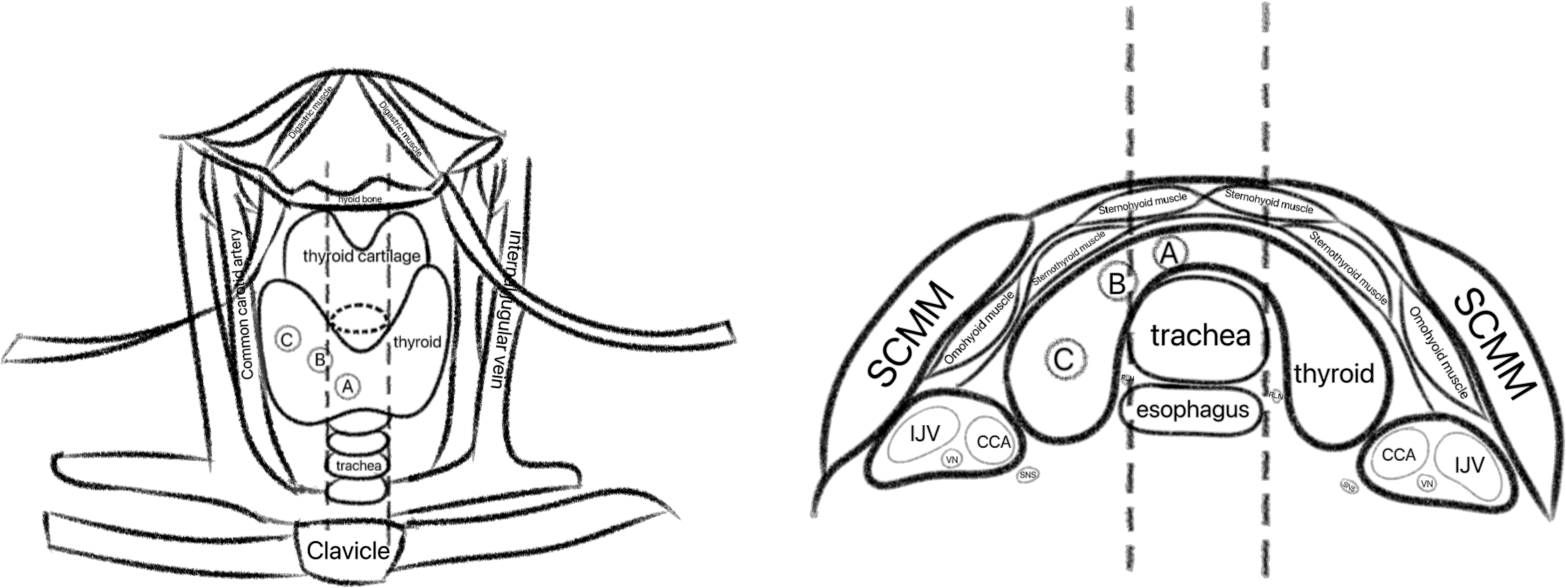
Schematic diagram illustrating PTC location classification based on CT imaging criteria. **(A)** Isthmus PTC: the tumor centroid lies between the projection lines drawn from the lateral tracheal margins. **(B)** Isthmus-proximity PTC: the centroid lies outside the projection lines while the medial tumor margin remains between them. **(C)** Non-isthmic (normally located) PTC: both the centroid and the medial tumor margin lie outside the projection lines.

Preoperative neck CT scans were used to identify the thyroid isthmus, the medial and lateral tumor margins, and the tumor centroid. The following definitions were applied: the tumor centroid was defined as the intersection of the tumor’s longest and shortest diameters; the thyroid isthmus was defined as the thyroid tissue anterior to the trachea connecting both thyroid lobes. Tumor location was classified according to the method described by Lim et al. (9): on axial CT images, vertical projection lines were drawn from the most lateral points of the trachea to the anterior skin surface. Tumors were categorized as: isthmus thyroid cancer, if the centroid fell between the two projection lines; isthmus-proximity PTC, if the centroid lay outside the projection lines but the medial tumor margin remained between them; or normally located thyroid cancer, if both the centroid and medial margin lay outside the projection lines (**Figure 1**). The central lymph node compartment was defined in accordance with the 2015 American Thyroid Association guidelines (10), which include the pretracheal, paratracheal, and prelaryngeal nodal groups. For statistical analysis, pretracheal and paratracheal lymph nodes were grouped and analyzed as part of the Ipsi-CLNs region.

### 2.2 Research subjects

This retrospective study enrolled patients with solitary PTC who underwent surgery at the Affiliated Hospital of Jiangsu University between January 2012 and June 2025. Inclusion criteria were: (1) postoperative pathological confirmation of PTC; (2) presence of a single tumor focus; (3) first-time surgery consisting of total or near-total thyroidectomy with bilateral central lymph node dissection; and (4) availability of complete preoperative imaging including thyroid ultrasound and neck CT. Exclusion criteria comprised: (1) tumors located in both lobes or the isthmus; (2) multifocal lesions within the same lobe; (3) previous history of other malignant neck tumors; (4) locally advanced PTC (stage T4a or higher) with invasion of critical structures such as the trachea, esophagus, prevertebral fascia, carotid artery, or mediastinal vessels; and (5) incomplete clinical or follow-up records that would preclude accurate analysis.

### 2.3 Data collection and image assessment

The following clinical and pathological variables were recorded and analyzed: age, gender, laterality of the tumor, presence of Hashimoto’s thyroiditis, BRAF V600E mutation status, findings from preoperative ultrasound and CT assessments, status of Ipsi-CLNs, aggressive pathological features (if present), tumor size, and extrathyroidal extension (ETE) (11).For preoperative imaging evaluation, lymph nodes were considered suspicious for metastasis on ultrasound based on established criteria: loss of the fatty hilum, cortical hyperechogenicity, calcifications, cystic changes, or abnormal peripheral vascularity (12). On CT, suspicion was raised by features including heterogeneous enhancement, necrosis or cystic degeneration, short-axis diameter ≥1.3 cm, clustering (≥3 nodes), irregular contours, indistinct margins, or fine granular calcifications (13).All ultrasound and CT images were reviewed independently by two radiologists, each with more than five years of subspecialty experience in thyroid imaging. Interobserver agreement was assessed. Any discrepancies in interpretation were resolved through a consensus review involving a third senior radiologist, whose judgment served as the final reference standard.

### 2.4 Model development and validation

Patients were first categorized as having isthmus-proximity or non−isthmic PTC based on tumor location, and the rates of contralateral central lymph node metastasis were compared between the two groups. From the isthmus−proximity cohort, patients were randomly divided into a training set (70%) and a test set (30%). Feature selection was performed within the training set using both LASSO regression and the Boruta algorithm. Variables identified by both methods were included in the final predictive model. Seven machine learning algorithms were implemented: logistic regression, decision tree, random forest, XGBoost, LightGBM, support vector machine (SVM), and artificial neural network (ANN). During model development, hyperparameters were optimized via five−fold cross−validation coupled with grid search, aiming to maximize the AUC. To address class imbalance, the synthetic minority oversampling technique (SMOTE) was applied separately within each cross−validation training fold; the corresponding validation fold remained unaltered to avoid data leakage. After hyperparameter tuning, each model was refitted on the entire training set and evaluated on the hold−out test set. Performance was assessed using accuracy, sensitivity, precision, F1−score, and AUC. For the best−performing model, SHAP (Shapley Additive Explanations) analysis was used to interpret feature importance. SHAP summary plots and dependence plots were generated to illustrate the direction and magnitude of each variable’s contribution to model predictions.

### 2.5 Statistical methods

Data were processed and analyzed using R (version 4.4.2). Variables with missing values exceeding 30% were excluded from the analysis. For the remaining variables with missing data, multiple imputation was performed using the mice package in R. Continuous variables with normal distribution are presented as mean ± standard deviation and were compared between groups using the independent samples t−test. Non−normally distributed continuous variables are reported as median (interquartile range) and were compared using the Mann−Whitney U test. Categorical variables are expressed as frequency (percentage) and were compared using the chi−square test or Fisher’s exact test, as appropriate. All statistical tests were two−sided, with a significance level set at α = 0.05.

## 3 Result

### 3.1 Baseline characteristics

**Figure 2:**
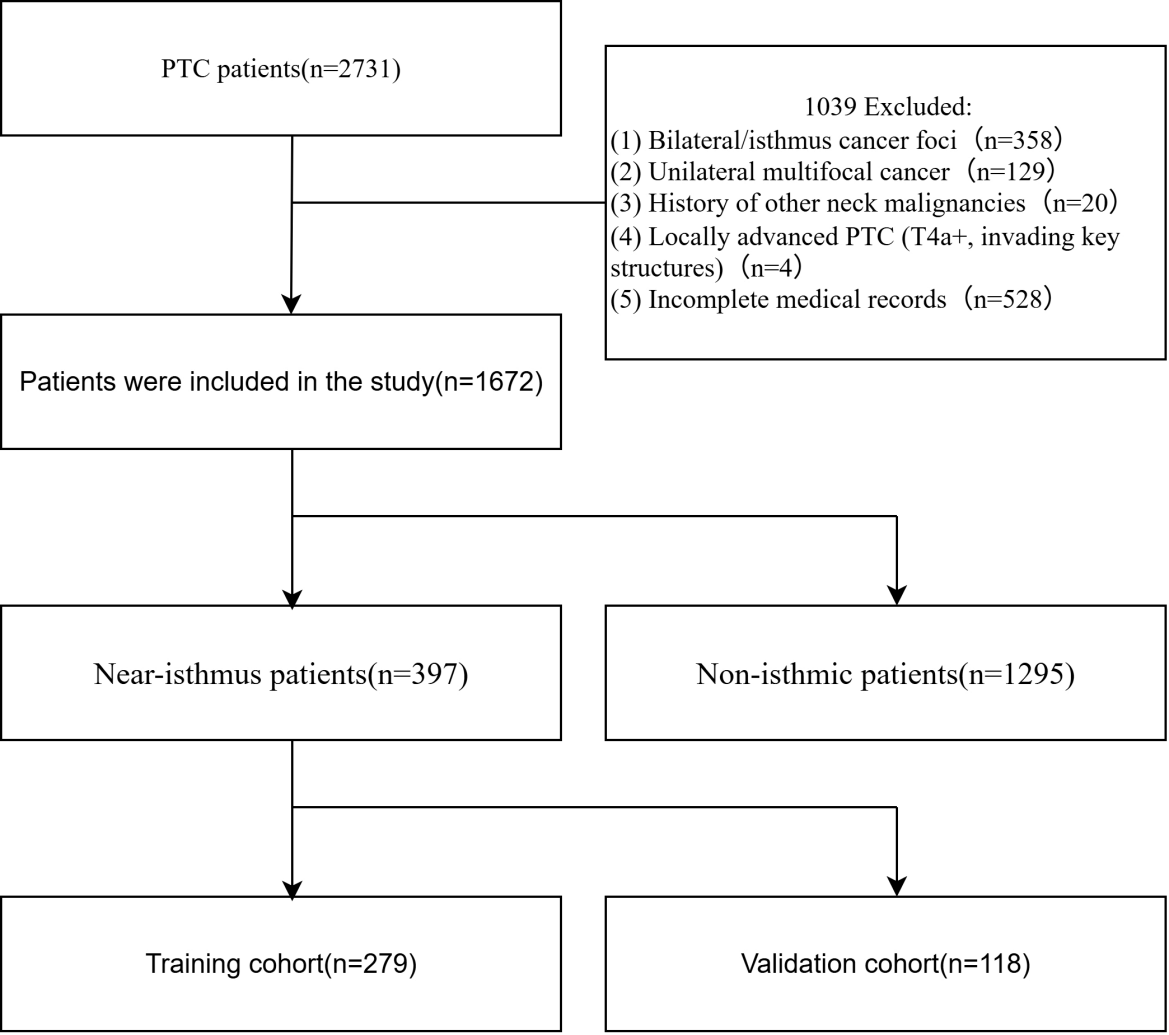
Patient flowchart for this study.

Between January 2012 and June 2025, a total of 2,711 patients with papillary thyroid carcinoma (PTC) were initially assessed. After applying the exclusion criteria, 1,672 patients were included in the final analysis (**Figure 2**). Reasons for exclusion were as follows: bilateral or isthmic PTC (n=358), unilateral multifocal carcinoma (n=129), previous history of neck malignancy (n=20), locally advanced disease (n=4), and incomplete clinical data (n=528). Of the 1,672 included patients, 397 were diagnosed with unilateral PTC with isthmus proximity, and the remaining had PTC in a typical (non-isthmic) location. The rate of Cont-CLNs metastasis was significantly higher in the isthmus-proximity group than in the non-isthmic group (*P* < 0.05; **Table 1**). These 397 patients with unilateral isthmus-proximity PTC were then randomly divided into a training set (n=278, 70%) and a test set (n=119, 30%) in a 7:3 ratio (**Table 2**). No statistically significant differences were observed in baseline characteristics between the training and test sets, confirming the comparability of the split.

**Table 1:** Metastasis patterns of PTC in the isthmus vs. unilateral normal position group.

Groups	Cont-CLNs		*χ²*	*P*
	Tumor metastasis	Tumor non-metastasis		
Normal group	153	1122	94.659	<0.001
Isthmus group	131	266		

**Table 2:** Clinical and pathological characteristics of patients in the training and validation sets.

Characteristics	Whole	Cont-CLNs		P	Cohort		P
Groups	Patients	No	Yes		Training	Validation	
n	397	266	131		279	118	
Age (median [IQR], years)	42.00 [33.00, 49.00]	42.00 [33.00, 50.00]	42.00 [32.50, 49.00]	0.334	42.00 [33.00, 50.00]	42.00 [33.25, 49.00]	0.469
Gender (male/female, %)	99/298(24.94/75.06)	60/206(22.56/77.44)	39/92(29.77/70.23)	0.15	68/211(24.37/75.63)	31/87(26.27/73.73)	0.689
Laterality (left/right, %)	205/192(51.64/48.36)	136/130(51.13/48.87)	69/62(52.67/47.33)	0.855	144/135(51.61/48.39)	61/57(51.69/48.31)	0.988
ETE (no/yes, %)	312/85(78.59/21.41)	247/19(92.86/7.14)	65/66(49.92/50.38)	<0.001	221/58(79.21/20.79)	91/27(77.12/22.88)	0.642
Ipsi (no/yes, %)	255/142(64.23/35.77)	206/60(77.44/22.56)	49/82(37.40/62.60)	<0.001	182/97(65.23/34.77)	73/45(61.86/38.14)	0.522
Pathology (no/yes,%)	372/25(93.70/6.30)	256/10(96.24/3.76)	116/15(88.55/11.45)	0.006	260/19(93.19/6.81)	112/6(94.92/5.08)	0.518
BRAF (no/yes, %)	22/375(5.54/94.46)	12/254(4.51/95.49)	10/121(7.63/92.37)	0.296	16/263(5.73/94.27)	6/112(5.08/94.92)	0.796
US (no/yes, %)	329/68(82.87/17.13)	252/14(94.74/5.26)	77/54(58.78/41.22)	<0.001	231/48(82.80/17.20)	98/20(83.05/16.95)	0.951
CT (no/yes, %)	319/78(80.35/19.65)	255/11(95.86/4.14)	64/67(48.85/51.15)	<0.001	223/56(79.93/20.07)	96/22(81.36/18.64)	0.744
HT(no/yes, %)	273/124(68.77/31.23)	186/80(69.92/30.08)	87/44(66.41/33.59)	0.552	198/81(70.97/29.03)	75/43(63.56/36.44)	0.145
Tumor Size (median [IQR], mm)	7.00 [6.00, 9.00]	6.00 [5.00, 8.00]	9.00 [7.00, 11.00]	<0.001	7.00 [6.00, 9.00]	7.00 [5.00, 9.00]	0.570

### 3.2 Feature selection: LASSO and Boruta

**Figure 3:**
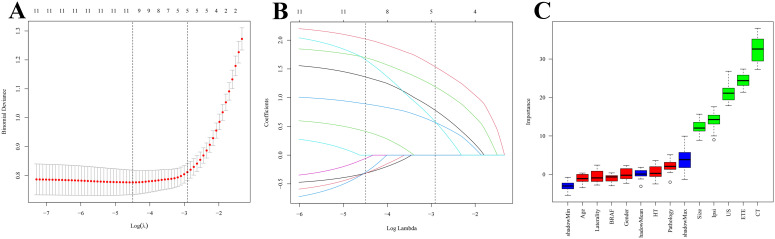
Lasso and Bourta screening. **(A)** Lasso log-lambda. **(B)** Lasso cross-validation. **(C)** Bourta screening.

The feature selection results from both LASSO regression and the Boruta algorithm consistently identified five key predictors: preoperative CT assessment, extrathyroidal extension, preoperative ultrasound assessment, Ipsi-CLNs metastasis, and tumor size (**Figure 3**).

### 3.3 Machine learning model evaluation

Based on the five selected predictors, seven machine learning algorithms were applied to build prediction models: logistic regression, decision tree, random forest, XGBoost, LightGBM, support vector machine, and artificial neural network. Model performance was systematically evaluated on the test set using the AUC, sensitivity, specificity, positive predictive value (PPV), negative predictive value (NPV), precision, recall, and F1-score (**Table 3**).

**Table 3:** Performance of seven models on the validation set.

Model	AUC	95% CI lower	95% CI upper	Accuracy	Precision	Sensitivity	Specificity	F1 score	Kappa	Youden’s J	PPV	NPV
Logistic	0.883	0.806	0.948	0.831	0.806	0.641	0.924	0.714	0.596	0.565	0.806	0.839
Decision Tree	0.840	0.750	0.918	0.839	0.813	0.667	0.924	0.732	0.619	0.591	0.813	0.849
Random Forest	0.861	0.772	0.940	0.864	0.848	0.718	0.937	0.778	0.681	0.655	0.848	0.871
XGBoost	0.880	0.804	0.946	0.831	0.913	0.538	0.975	0.677	0.573	0.513	0.913	0.811
LightGBM	0.881	0.805	0.945	0.822	0.781	0.641	0.911	0.704	0.579	0.552	0.781	0.837
SVM	0.827	0.726	0.916	0.805	0.682	0.769	0.823	0.723	0.573	0.592	0.682	0.878
ANN	0.882	0.805	0.947	0.830	0.806	0.641	0.924	0.714	0.596	0.565	0.806	0.839

**Figure 4:**
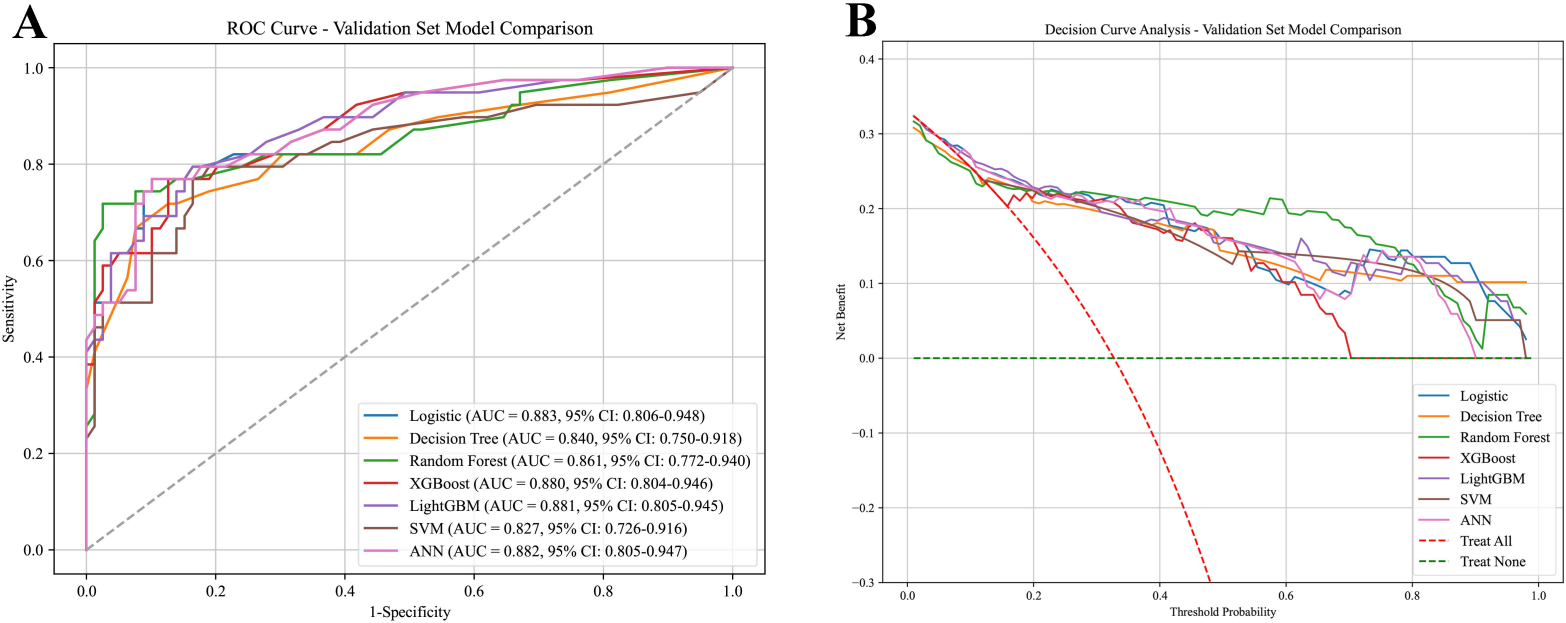
ROC curves and DCA curves for each model. **(A)** ROC curves for each model. **(B)** DCA curves for each model.

The random forest model showed the best overall performance, attaining the highest F1-score and AUC values of 0.942 in the training set and 0.861 in the validation set. Furthermore, decision curve analysis confirmed its favorable clinical net benefit across a range of threshold probabilities, supporting its practical utility in clinical decision-making (**Figure 4**).

### 3.4 Model interpretability analysis

**Figure 5:**
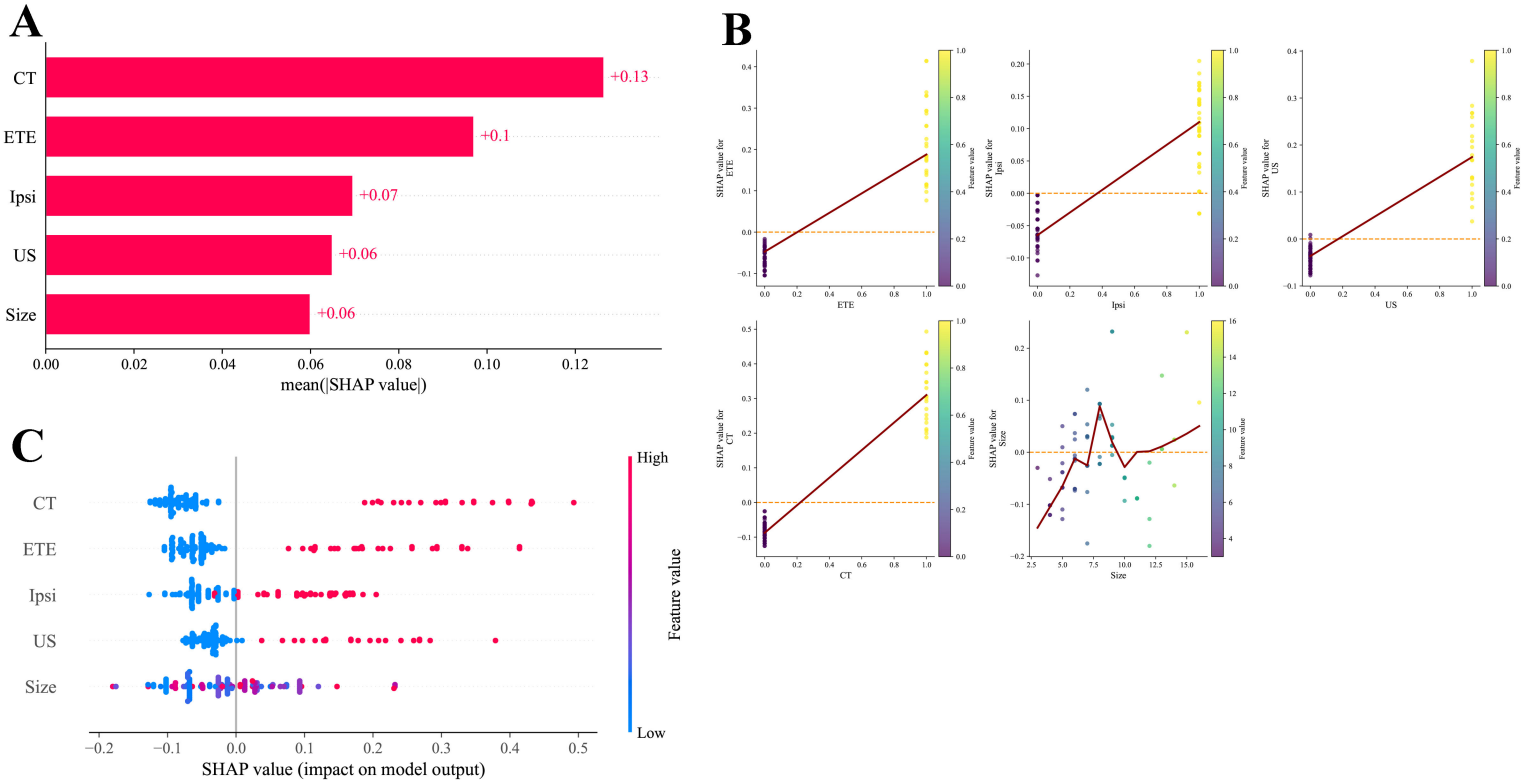
SHAP analysis results based on the RF model. **(A)** SHAP feature ranking plot. **(B)** SHAP feature honeycomb plot. **(C)** SHAP dependency plot.

To interpret how the random forest model predicts Cont-CLNs metastasis in patients with isthmus-proximity PTC, SHAP analysis was performed. The five most influential predictors, ranked in descending order of SHAP importance, were: preoperative CT assessment, extrathyroidal extension, Ipsi-CLNs metastasis, preoperative ultrasound assessment, and tumor size (**Figure 5A**). This order suggests that imaging and pathological markers of local invasion and nodal involvement carry greater predictive weight than tumor size alone.

SHAP summary and dependence plots (**Figures 5B, C**) illustrated the direction and magnitude of each feature’s effect. A clear “dose–response” pattern was observed for preoperative CT assessment: negative findings corresponded to negative SHAP values, lowering the predicted probability of metastasis, whereas positive findings produced strongly positive SHAP values, making this the strongest driver of the model’s risk prediction. Extrathyroidal extension and Ipsi-CLNs metastasis showed a similar directional influence, with presence of either feature substantially increasing predicted risk, though the effect of Ipsi-CLNs was slightly weaker. Preoperative ultrasound assessment followed the same trend, with positive findings raising the estimated probability of metastasis. By contrast, tumor size exhibited a more complex, nonlinear relationship with predicted risk. Although larger tumors (especially >10 mm) were weakly associated with increased risk, size alone was not a stable or strong independent predictor in this model. These interpretability findings align closely with established clinical understanding, supporting the biologic plausibility and practical relevance of the model.

## 4 Discussion

PTC, the most common malignant tumor of the thyroid, generally carries a favorable prognosis. However, it has a propensity for early lymphatic metastasis, particularly to the central neck compartment. In addition to Ipsi-CLNs involvement, Cont-CLNs metastasis is also clinically significant. In this study, the Cont-CLNs metastasis rate reached 33% among patients with unilateral PTC located near the thyroid isthmus. Currently, ultrasound and neck CT exhibit relatively low sensitivity for preoperative detection of Cont-CLNs metastasis (14), meaning that only a subset of patients are accurately diagnosed prior to surgery. Omitting Cont-CLNs dissection during initial surgery substantially increases the technical difficulty of revision procedures and raises the risk of complications such as recurrent laryngeal nerve injury and hypoparathyroidism. Thus, developing an accurate predictive model for Cont-CLNs metastasis is clinically valuable, as it may inform the extent of lymph node dissection during first-time surgery.

In this study, a prediction model was developed using machine learning, and after comprehensive evaluation, the random forest algorithm was selected as optimal. The model demonstrated strong predictive performance, with AUC values of 0.942 in the training set and 0.861 in the validation set. Interpretability analysis using SHAP showed close alignment with clinical reasoning: preoperative CT and ultrasound assessments emerged as highly important features, highlighting the central role of imaging in surgical planning. Furthermore, extrathyroidal extension and Ipsi-CLNs metastasis—both strong predictors—reinforce the concept that local invasiveness and regional nodal spread are key determinants of contralateral metastasis. By quantifying the contribution of individual predictors, the model offers a basis for personalized surgical strategy. For instance, patients with positive preoperative CT findings, extrathyroidal extension, or Ipsi-CLNs metastasis receive higher composite risk scores and may be considered for more comprehensive Cont-CLNs dissection. Such a transparent risk-stratification tool may help clinicians balance the risks of under- and over-treatment.

Although preoperative CT and ultrasound are limited in sensitivity for Cont-CLNs metastasis, they offer high specificity, and the combination of contrast-enhanced CT with ultrasound is particularly useful for identifying suspicious lymph nodes. Therefore, routine preoperative neck CT and ultrasound are recommended in thyroid cancer patients to thoroughly evaluate local invasion and nodal disease, thereby guiding the selection of surgical approach (15).

Extrathyroidal extension in thyroid cancer is associated with greater aggressiveness and a higher likelihood of lymph node metastasis (16). Isthmic PTC has been reported to show higher rates of bilateral central neck metastasis compared with unilateral non-isthmic PTC (17), possibly due to its inherently more invasive phenotype and greater propensity for extrathyroidal extension (18). In the present study, the rate of extrathyroidal extension was significantly higher in the isthmus-proximity PTC group than in the non-isthmic group, and the Cont-CLNs metastasis rate was also significantly different between the two groups. This suggests that PTC situated near the isthmus may share biological behavior with true isthmic PTC, thereby conferring an elevated risk of Cont-CLNs metastasis.

Multiple studies have identified Ipsi-CLNs metastasis as a risk factor for Cont-CLNs metastasis (19), a finding consistent with our results. The underlying mechanism may involve lymphatic communications between the bilateral central compartments (20). Therefore, in patients with isthmus-proximity PTC, careful assessment of Ipsi-CLNs status is warranted. If intraoperative suspicion of metastasis exists, frozen-section analysis could help determine the need for Cont-CLNs dissection (21). In addition, tumor size is a recognized predictor of Ipsi-CLNs metastasis (22), a notion partly supported by our data: for tumors >10 mm, the risk of Cont-CLNs metastasis tended to increase with increasing tumor size.

Whether to perform bilateral central neck dissection in unilateral PTC remains debated, largely due to concerns over complications such as bilateral recurrent laryngeal nerve palsy and permanent hypoparathyroidism. However, advances such as nanocarbon tracing, intraoperative nerve monitoring, and intraoperative parathyroid hormone assay—coupled with refined surgical technique and heightened awareness of anatomical preservation—have substantially lowered complication rates. For patients with unilateral isthmus-proximity PTC, the proposed prediction model can help estimate the risk of Cont-CLNs metastasis. If the predicted risk is high, bilateral central neck dissection may be recommended.

Several limitations of this study should be noted. First, its single-center retrospective design and relatively modest sample size may affect generalizability. Future multicenter studies with larger, more diverse cohorts are needed to improve model robustness and external validity. Second, confirmation of Ipsi-CLNs metastasis relied on intraoperative frozen section, which is not universally available and can prolong operative time. Third, preoperative CT and ultrasound assessments are subject to inter-observer variability. Developing more objective, standardized imaging criteria—potentially assisted by artificial intelligence-based tools—would be valuable. Although some predictive models already exist (23, 24), further validation in broader clinical settings is necessary.

## Data Availability

The original contributions presented in the study are included in the article/**Supplementary Material**. Further inquiries can be directed to the corresponding author.

## References

[1] Huang J Ngai CH Deng Y Pun CN Lok V Zhang L . Incidence and mortality of thyroid cancer in 50 countries: a joinpoint regression analysis of global trends. Endocrine. (2023) 80:355–65. doi: 10.1007/s12020-022-03274-7, PMID: 36607509

[2] Boucai L Zafereo M Cabanillas ME . Thyroid cancer: A review. Jama. (2024) 331:425–35. doi: 10.1001/jama.2023.26348, PMID: 38319329

[3] Alsubaie KM Alsubaie HM Alzahrani FR Alessa MA Abdulmonem SK Merdad MA . Prophylactic central neck dissection for clinically node-negative papillary thyroid carcinoma. Laryngoscope. (2022) 132:1320–8. doi: 10.1002/lary.29912, PMID: 34708877

[4] Chen DW Lang BHH McLeod DSA Newbold K Haymart MR . Thyroid cancer. Lancet (London England). (2023) 401:1531–44. doi: 10.1016/S0140-6736(23)00020-X, PMID: 37023783

[5] Kim DH Kim GJ Kim SW Hwang SH . Predictive value of ipsilateral central lymph node metastasis for contralateral central lymph node metastasis in patients with thyroid cancer: Systematic review and meta-analysis. Head Neck. (2021) 43:3177–84. doi: 10.1002/hed.26787, PMID: 34124791

[6] Chen ZH Shui CY Wang AL Xu N Hu BT Sun RH . Influencing factors and predictive model development for contralateral central lymph node metastasis in unilateral papillary thyroid microcarcinoma. Zhonghua yi xue za zhi. (2025) 105:1607–13. doi: 10.3760/cma.j.cn112137-20241216-02837, PMID: 40399125

[7] Liu N Chen B Li L Zeng Q Sheng L Zhang B . Effect of tumor location on the risk of bilateral central lymph node metastasis in unilateral 1–4 cm papillary thyroid carcinoma. Cancer Manage Res. (2021) 13:5803–12. doi: 10.2147/CMAR.S318076, PMID: 34321927 PMC8312608

[8] Li Y Lao L . Comparison of prophylactic ipsilateral and bilateral central lymph node dissection in papillary thyroid carcinoma: a meta-analysis. Braz J Otorhinolaryngol. (2023) 89:101318. doi: 10.1016/j.bjorl.2023.101318, PMID: 37716097 PMC10509659

[9] Zhao J Zhang Y Zheng X . Clinicopathological characteristics of papillary thyroid cancer located in the isthmus with Delphian lymph node metastasis. Br J Oral Maxillofac Surg. (2022) 60:635–8. doi: 10.1016/j.bjoms.2021.11.016, PMID: 35210104

[10] Haugen BR Alexander EK Bible KC Doherty GM Mandel SJ Nikiforov YE . 2015 American thyroid association management guidelines for adult patients with thyroid nodules and differentiated thyroid cancer: the American thyroid association guidelines task force on thyroid nodules and differentiated thyroid cancer. Thyroid: Off J Am Thyroid Assoc. (2016) 26:1–133. doi: 10.1089/thy.2015.0020, PMID: 26462967 PMC4739132

[11] Zhou W Li L Hao X Wu L Liu L Zheng B . Predicting central lymph node metastasis in papillary thyroid microcarcinoma: a breakthrough with interpretable machine learning. Front Endocrinol. (2025) 16:1537386. doi: 10.3389/fendo.2025.1537386, PMID: 40421246 PMC12104047

[12] Wang X Qi Y Zhang X Liu F Li J . Ultrasound-based artificial intelligence for predicting cervical lymph node metastasis in papillary thyroid cancer: a systematic review and meta-analysis. Front Endocrinol. (2025) 16:1570811. doi: 10.3389/fendo.2025.1570811, PMID: 40556829 PMC12185295

[13] Roh YH Chung SR Yang SJ Baek JH Choi YJ Sung TY . Enhancement on CT for preoperative diagnosis of metastatic lymph nodes in thyroid cancer: a comparison across experience levels. Eur Radiol. (2025) 35:20–8. doi: 10.1007/s00330-024-10919-w, PMID: 38980412

[14] Albuck AL Issa PP Hussein M Aboueisha M Attia AS Omar M . A combination of computed tomography scan and ultrasound provides optimal detection of cervical lymph node metastasis in papillary thyroid carcinomas: A systematic review and meta-analysis. Head Neck. (2023) 45:2173–84. doi: 10.1002/hed.27451, PMID: 37417426

[15] Alabousi M Alabousi A Adham S Pozdnyakov A Ramadan S Chaudhari H . Diagnostic test accuracy of ultrasonography vs computed tomography for papillary thyroid cancer cervical lymph node metastasis: A systematic review and meta-analysis. JAMA Otolaryngol Head Neck Surg. (2022) 148:107–18. doi: 10.1001/jamaoto.2021.3387, PMID: 34817554 PMC8613701

[16] Shin CH Roh JL Song DE Cho KJ Choi SH Nam SY . Prognostic value of tumor size and minimal extrathyroidal extension in papillary thyroid carcinoma. Am J Surg. (2020) 220:925–31. doi: 10.1016/j.amjsurg.2020.02.020, PMID: 32081409

[17] Lee YC Na SY Chung H Kim SI Eun YG . Clinicopathologic characteristics and pattern of central lymph node metastasis in papillary thyroid cancer located in the isthmus. Laryngoscope. (2016) 126:2419–21. doi: 10.1002/lary.25926, PMID: 27098428

[18] Karatzas T Charitoudis G Vasileiadis D Kapetanakis S Vasileiadis I . Surgical treatment for dominant malignant nodules of the isthmus of the thyroid gland: A case control study. Int J Surg (London England). (2015) 18:64–8. doi: 10.1016/j.ijsu.2015.04.039, PMID: 25900600

[19] Chen Q Liu Y Lu W Zhang L Su A Liu F . Pretracheal lymph node subdivision in predicting contralateral central lymph node metastasis for unilateral papillary thyroid carcinoma: preliminary results. Front Endocrinol. (2022) 13:921845. doi: 10.3389/fendo.2022.921845, PMID: 35923620 PMC9339796

[20] Bohec H Breuskin I Hadoux J Schlumberger M Leboulleux S Hartl DM . Occult contralateral lateral lymph node metastases in unilateral N1b papillary thyroid carcinoma. World J Surg. (2019) 43:818–23. doi: 10.1007/s00268-018-4862-9, PMID: 30465086

[21] Chae BJ Jung CK Lim DJ Song BJ Kim JS Jung SS . Performing contralateral central lymph node dissection in papillary thyroid carcinoma: a decision approach. Thyroid: Off J Am Thyroid Assoc. (2011) 21:873–7. doi: 10.1089/thy.2010.0214, PMID: 21745104

[22] Kim SK Park I Hur N Rayzah M Lee JH Choe JH . Patterns, predictive factors, and prognostic impact of contralateral lateral lymph node metastasis in N1b papillary thyroid carcinoma. Ann Surg Oncol. (2017) 24:1943–50. doi: 10.1245/s10434-016-5761-7, PMID: 28160142

[23] Du J He X Fan R Zhang Y Liu H Liu H . Artificial intelligence-assisted precise preoperative prediction of lateral cervical lymph nodes metastasis in papillary thyroid carcinoma via a clinical-CT radiomic combined model. Int J Surg (London England). (2025) 111:2453–66. doi: 10.1097/JS9.0000000000002267, PMID: 39903541 PMC12372748

[24] Feng JW Yang YX Qin RJ Liu SQ Qin AC Jiang Y . Application and validation of the machine learning-based multimodal radiomics model for preoperative prediction of lateral lymph node metastasis in papillary thyroid carcinoma. Front Endocrinol. (2025) 16:1618902. doi: 10.3389/fendo.2025.1618902, PMID: 40904800 PMC12401691

