# Supplementary figures and images for: Machine learning-based model for predicting contralateral central lymph node metastasis in papillary thyroid carcinoma with isthmus proximity
Source: Front Endocrinol (Lausanne). 2026 Jan 9;16:1728945. doi: 10.3389/fendo.2025.1728945 (PMC12827097; doi:10.3389/fendo.2025.1728945)

Decision Curve Analysis - Training Set Model Comparison

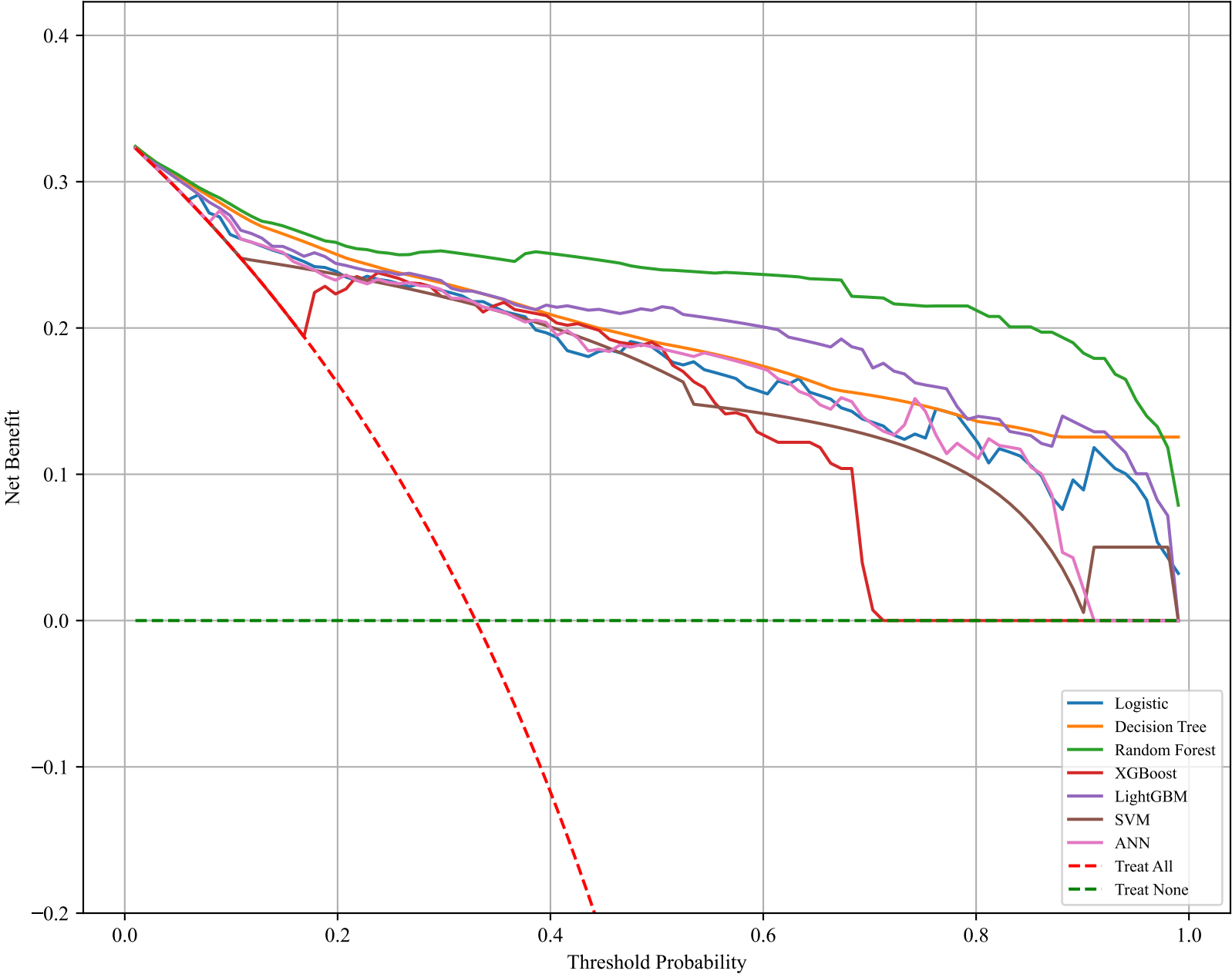

Supplement: Supplementary file 1 [file DataSheet1.pdf]

ROC Curve - Training Set Model Comparison

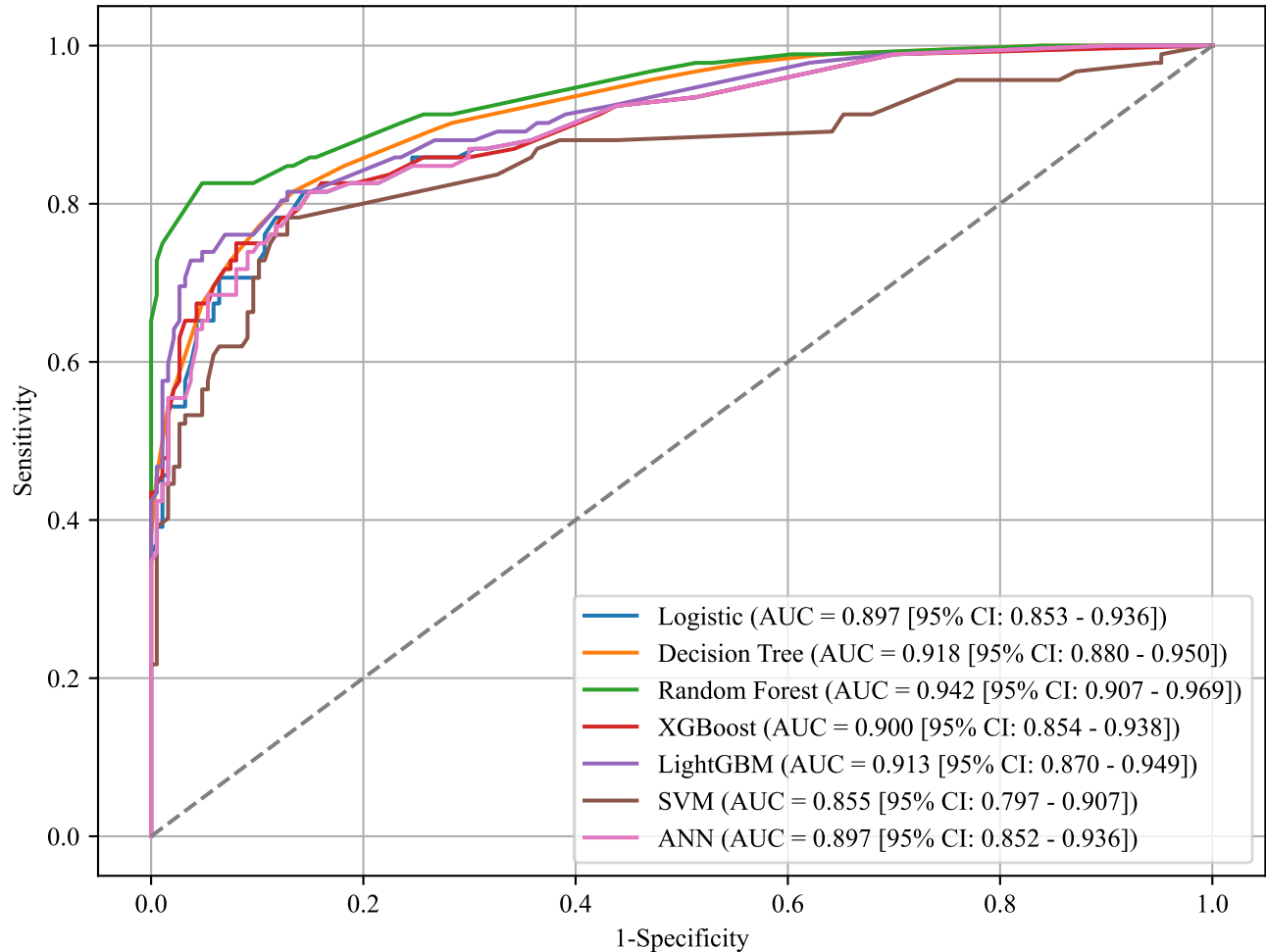

Supplement: Supplementary file 4 [file DataSheet4.pdf]
