# Supplementary material for: Machine learning-based model for predicting contralateral central lymph node metastasis in papillary thyroid carcinoma with isthmus proximity
Source: Front Endocrinol (Lausanne). 2026 Jan 9;16:1728945. doi: 10.3389/fendo.2025.1728945 (PMC12827097; doi:10.3389/fendo.2025.1728945)
Supplement: Supplementary file 2 [file DataSheet2.pdf]

Detailed Parameters of the Optimized Decision Tree Model: 0

ccp_alpha	0.0
class_weight	None
criterion	gini
max_depth	10
max_features	None
max_leaf_nodes	None
min_impurity_decrease	0.0
min_samples_leaf	1
min_samples_split	20
min_weight_fraction_leaf	0.0
monotonic_cst	None
random_state	123
splitter	best

Detailed parameters of the optimized RF model: 0

bootstrap	True
ccp_alpha	0.0
class_weight	None
criterion	gini
max_depth	None
max_features	2
max_leaf_nodes	None
max_samples	None
min_impurity_decrease	0.0
min_samples_leaf	1
min_samples_split	2
min_weight_fraction_leaf	0.0
monotonic_cst	None
n_estimators	200
n_jobs	None
oob_score	True
random_state	123
verbose	0
warm_start	False

Detailed parameters of the tuned XGBoost model: 0

objective	binary:logistic
base_score	None
booster	None
callbacks	None
colsample_bylevel	None
colsample_bynode	None

colsample_bytree	None
device	None
early_stopping_rounds	None
enable_categorical	False
eval_metric	logloss
feature_types	None
feature_weights	None
gamma	None
grow_policy	None
importance_type	None
interaction_constraints	None
learning_rate	0.01
max_bin	None
max_cat_threshold	None
max_cat_to_onehot	None
max_delta_step	None
max_depth	3
max_leaves	None
min_child_weight	None
missing	NaN
monotone_constraints	None
multi_strategy	None
n_estimators	100
n_jobs	None
num_parallel_tree	None
random_state	123
reg_alpha	None
reg_lambda	None
sampling_method	None
scale_pos_weight	None
subsample	0.6
tree_method	None
validate_parameters	None
verbosity	None

Detailed Parameters for Optimizing LightGBM Models: 0

boosting_type	gbdt
class_weight	None
colsample_bytree	0.6
importance_type	split
learning_rate	0.1
max_depth	-1
min_child_samples	20
min_child_weight	0.001

min_split_gain	0.0
n_estimators	100
n_jobs	None
num_leaves	31
objective	None
random_state	123
reg_alpha	0.0
reg_lambda	0.0
subsample	0.6
subsample_for_bin	200000
subsample_freq	0

Detailed Parameters for Optimizing SVM Models:	0
C	0.1
break_ties	False
cache_size	200
class_weight	None
coef0	0.0
decision_function_shape	ovr
degree	2
gamma	scale
kernel	linear
max_iter	-1
probability	True
random_state	123
shrinking	True
tol	0.001
verbose	False

Detailed Parameters for Optimizing ANN Models:	0
activation	logistic
alpha	0.0001
batch_size	auto
beta_1	0.9
beta_2	0.999
early_stopping	False
epsilon	0.0
hidden_layer_sizes	(10, 10)
learning_rate	constant
learning_rate_init	0.001
max_fun	15000

max_iter	500
momentum	0.9
n_iter_no_change	10
nesterovs_momentum	True
power_t	0.5
random_state	123
shuffle	True
solver	adam
tol	0.0001
validation_fraction	0.1
verbose	False
warm_start	False
